# Strategic Superposition and Replicator Dynamics: Quantum Collapses in Decision Processes

**DOI:** 10.3390/e28070827

**Published:** 2026-07-20

**Authors:** Aras Yolusever

**Affiliations:** Department of Economics, Faculty of Economics and Administrative Sciences, Istanbul Kültür University, Küçükçekmece Campus, Küçükçekmece, 34307 Istanbul, Türkiye; a.yolusever@iku.edu.tr

**Keywords:** quantum decision theory, evolutionary game theory, replicator dynamics, strategic superposition, behavioral economics

## Abstract

Classical evolutionary game theory rests on a hidden assumption: that an actor holds a definite strategy, pure or randomized, before it ever interacts. Yet human and organizational choices routinely violate this premise, displaying interference, order, and framing effects that classical probability cannot accommodate. We propose a framework in which an economic actor is genuinely undecided before market entry, modeled as a strategic superposition of pure strategies in a Hilbert space, and in which interaction acts as a measurement that collapses this state onto a realized strategy with Born-rule probabilities. Populations are described by a density operator whose diagonal carries strategy frequencies and whose off-diagonal coherences encode maintained superposition, evolving under a strategic master equation that couples coherent deliberation, decoherence in the strategy basis, and a replicator selection superoperator. Three results follow. A square-root representation places quantum normalization and evolutionary selection on a common geometric footing; the classical replicator equation emerges exactly as the strong-decoherence limit, with an explicit error bound; and strategy realization becomes basis-dependent through interference that no classical mixture reproduces. In a two-strategy market game, coherent coupling displaces the evolutionarily stable strategy by order Δ^2^/γ, recovering the classical value as decoherence dominates. The construction formalizes constitutive self-opacity and links bounded rationality to quantum interference, positioning classical evolutionary dynamics as one limiting regime of a broader strategic dynamics.

## 1. Introduction

Game theory studies strategic interaction, the situations in which the outcome for each actor depends on the choices of all. Since its formalization by von Neumann and Morgenstern [1] and the equilibrium analysis of Nash [2], it has become a common language across economics, biology, and computer science for reasoning about conflict and cooperation among rational agents. Evolutionary game theory extends this apparatus from one-shot rational choice to populations of interacting agents whose strategy frequencies change over time under selection, replacing the demand for full rationality with a dynamic of differential success. It is within this evolutionary branch that the present work is situated.

Evolutionary game theory inherited from its biological origins a sharp ontology of strategy. An actor either plays a pure strategy, drawn from a finite repertoire, or plays a mixed strategy understood as a classical probability distribution over that repertoire [3,4]. Population-level change is then described by the replicator equation, which raises the frequency of strategies whose payoff exceeds the population average [5,6,7]. In both the pure and the mixed reading, the actor is taken to hold a strategy before interaction occurs. Randomization, where present, is a classical lottery: the actor possesses a definite probability vector, and the only uncertainty concerns which sample is realized.

A growing body of behavioral evidence sits uneasily with this picture. Choices display order effects, disjunction effects, and framing effects that violate the axioms of classical probability and expected utility [8,9]. Quantum decision theory responds by replacing classical probability with the probability calculus of quantum mechanics, in which amplitudes rather than probabilities are the primitive objects and interference between alternatives becomes possible [10,11,12,13,14,15,16]. In parallel, the quantum games program quantizes the act of play itself, allowing actors to select unitary operations on entangled qubits and studying the resulting equilibria [17,18,19,20].

Neither line of work addresses the question this paper takes up. Quantum decision theory models the single choice of a single agent and stops at the moment of decision. The quantum games program quantizes strategies and payoffs but works with equilibrium concepts rather than population dynamics, and it does not treat the pre-decision state of an actor as an evolving object subject to selection. What is missing is a framework in which (a) an actor is genuinely undecided before interaction, in the strong sense that no definite strategy exists rather than the weak sense that an existing strategy is unknown; (b) interaction collapses this indeterminate state onto a realized strategy; and (c) the distribution of realized strategies, together with the underlying amplitudes, is reshaped over evolutionary time by differential fitness.

The objective of this paper is to construct and analyze a single dynamical framework in which the pre-interaction strategic state is genuinely indeterminate, collapses to a realized strategy at the moment of interaction, and is reshaped by selection over evolutionary time, and to determine the precise conditions under which this quantum strategic dynamics reduces to classical replicator dynamics. This paper supplies that framework. We model the firm before market entry as a superposition of pure strategies, treat market interaction as a measurement that collapses the superposition, and couple the resulting frequencies to replicator dynamics through a density-operator master equation. The construction has three payoffs beyond unification. First, it gives an exact sense in which classical evolutionary dynamics is a limiting regime of a more general quantum strategic dynamics, recovered when interaction with the environment is strongly decohering. Second, it predicts that the realized strategy distribution of a superposed firm depends on the context, or measurement basis, in which the interaction is framed, a dependence that no classical mixed strategy with the same marginals can reproduce. Third, it formalizes the notion of constitutive self-opacity that anchors the Homo Evolutivus program: the firm’s strategy is constitutively indefinite, not merely hidden from observers or from the firm itself, and superposition is the natural mathematics of that indefiniteness.

### Related Work

Game theory originates in the work of von Neumann and Morgenstern [1] and acquired its central solution concept, the equilibrium in which no player can profitably deviate, with Nash [2]. Evolutionary game theory reinterpreted equilibrium as the rest point of a population process rather than the deduction of ideal reasoners: Maynard Smith and Price introduced the evolutionarily stable strategy [3], and Taylor and Jonker the replicator dynamics [5], with the mathematical theory consolidated by Hofbauer and Sigmund [6,21], Weibull [7], and Sandholm [22]. Recent surveys document a renewed cross-disciplinary interest in these dynamics and their behavioral foundations [23]. Throughout this tradition, the strategy set is classical: an actor plays a pure strategy or a classical mixture, and the population state is a point of the probability simplex.

A parallel literature documents systematic departures of human and organizational choice from classical probability and expected utility, including disjunction, order, and framing effects [8,9]. Quantum decision theory and quantum cognition respond by adopting the probability calculus of quantum theory, in which amplitudes rather than probabilities are primitive and interference between alternatives becomes possible. This approach has been developed into models of individual judgment and choice by Busemeyer and Bruza [10], Pothos and Busemeyer [11,12], Yukalov and Sornette [13], Aerts [14], and Khrennikov and coauthors [15,16], and is surveyed by Bruza, Wang, and Busemeyer [24]. This body of work models the single decision of a single agent and typically stops at the moment of choice, without embedding the decision in a population that evolves under selection.

A distinct program quantizes the act of play itself. Beginning with Meyer [17] and Eisert, Wilkens, and Lewenstein [18], and continued by Marinatto and Weber [19] and Piotrowski and Sładkowski [20], quantum game theory allows players to choose unitary operations on entangled states and studies the resulting equilibria; the history and interpretation of the field are reviewed by Khan, Solmeyer, Balu, and Humble [25]. These models generalize the strategy space and the equilibrium concept, but they remain within a static, equilibrium-centered analysis and do not treat the pre-decision strategic state as an object that evolves over time.

Closest to the present work are the few studies that connect quantum structure to evolutionary dynamics. Iqbal and Toor asked whether a small group of mutants playing quantum strategies can invade a classical evolutionarily stable strategy, and showed that entanglement can create or destroy evolutionary stability without altering the underlying Nash equilibria [26]. Guevara Hidalgo proposed a quantum replicator dynamics by identifying the population state with a density operator evolving under a von Neumann type equation drawn from quantum statistical mechanics [27]. Our construction differs from both in its object and its mechanism. Rather than asking whether quantum strategies invade a fixed equilibrium, or positing a density-operator analog of selection by formal analogy, we model the pre-interaction firm as a genuine strategic superposition, treat market interaction as a measurement that collapses it with Born-rule probabilities, and couple the resulting frequencies to replicator selection through a single master equation that also contains decoherence in the strategy basis. This yields the classical replicator equation as an explicit strong-decoherence limit (Theorem A1), a positivity guarantee for the nonlinear dynamics (Proposition A3), and a basis dependence of realized strategies that no classical mixture reproduces (Proposition A2), none of which appears in the prior quantum-evolutionary literature.

The gap this paper addresses is therefore specific. Quantum decision theory models a single agent’s single choice and stops at the moment of decision; the quantum games program quantizes strategies and payoffs but remains within static equilibrium analysis; and classical evolutionary game theory evolves populations but presupposes definite pre-interaction strategies. No existing framework treats the pre-decision strategic state as genuinely indeterminate, collapses it at interaction, and couples the resulting frequencies to selection over evolutionary time. The novelty of this work is to close that gap with a single density-operator dynamics. Our main contributions are threefold: we derive a square-root representation unifying quantum normalization and evolutionary selection (Proposition A1); we prove that classical replicator dynamics emerges as the strong-decoherence limit with an explicit error bound and an established positivity guarantee (Theorem A1, Proposition A3); and we show that strategy realization is basis dependent in a way no classical mixture can reproduce (Proposition A2). Together, these results position classical evolutionary dynamics as one limiting regime of a broader quantum strategic dynamics and give formal content to the notion of constitutive self-opacity.

## 2. Theoretical Framework

### 2.1. The Strategy Hilbert Space and Basis States

Let an actor face a finite set of pure strategies $S\;=\;\{s_{1},s_{2},\ldots ,s_{n}\}$ We associate with S a complex Hilbert space $H\;≅{\;C}^{n}$ and a distinguished orthonormal basis $\left\{\left|s_{1}\rangle ,\ldots ,\right|s_{n}\rangle \right\}$ the strategy basis, with $\langle s_{i}|s_{j}\rangle \;=\;\delta_{\mathrm{ij}}$. A committed actor playing the pure strategy $s_{i}$ is in the basis state $\left|s_{i}\right\rangle$. The strategy basis plays the role of the pointer basis in the measurement theory of open systems: it is the set of outcomes that interaction with the market renders definite [28].

### 2.2. Superposition, the Born Rule, and Collapse at Interaction

An actor who has not committed to a pure strategy is in a superposition

(1) $$\left|\psi \rangle \;=\sum_{i=1}^{n}c_{i}\;\right|s_{i}\rangle ,\;\;c_{i}\in C,\;\;\sum_{i=1}^{n}{\left|c_{i}\right|}^{2}=1.$$

In Equation (1), the complex numbers $c_{i}$ are probability amplitudes. They carry more information than a classical probability vector, since each amplitude has a modulus and a phase, $c_{i\;}={\;r}_{i}e^{i\theta_{i}}$ with $r_{i\;}\geq \;0$. The squared moduli reproduce a classical distribution over strategies, while the phases encode relations between strategies that have no classical counterpart and become observable only under a change in measurement context or under entanglement.

When the actor interacts with the market in the strategy basis, the realized strategy is $s_{i}$ with probability given by the Born rule [29], as in Equation (2)

(2) Prsi=⟨siψ⟩=2ci|2=ri2.

We write $p_{i}:={\left|c_{i}\right|}^{2}$ for the realization probability of $s_{i}$. The normalization $\sum_{i}{\left|c_{i}\right|}^{2}=1$ guarantees that $\left(p_{1},\ldots ,p_{n}\right)$ is a point of the probability simplex $\Delta^{n-1}$.

The conceptual core of the model is the identification of the moment of interaction with a measurement. Before the firm enters the market, plays the game, or commits resources, its strategic state is the superposition |ψ⟩. Entry is an observation in the strategy basis, described by the projective (von Neumann) measurement with projectors $P_{i}\;=\;\left|s_{i}\rangle \langle s_{i}\right|$ [30]. The interaction yields outcome $s_{i}$ with probability $p_{i}\;=\;\langle \psi \left|P_{i}\right|\psi \rangle ={\left|c_{i}\right|}^{2}$, and the post-interaction state collapses to the corresponding basis state, $\left|\psi \rangle \to \right|s_{i}\rangle$ with probability ${\left|c_{i}\right|}^{2}$. The realized strategy then earns the payoff determined by the game. Two features deserve emphasis. The strategy that is realized is created at the moment of interaction; it is not read off a value the firm secretly held. And the probabilities that govern realization are exactly the squared amplitudes, so the act of collapse generates the very frequencies whose population-level evolution the replicator dynamics will govern.

Projective measurement is an idealization in which interaction extracts complete information and produces full commitment. Markets frequently do less. A firm may signal partial intent, leak partial information, or commit gradually. The appropriate generalization is a positive operator-valued measure, a family of positive operators $\left\{E_{k}\right\}$ with $\sum_{k}E_{k}\;=\;I$ [31]. Outcome k occurs with probability $\mathrm{Pr}\left(k\right)\;=\;\langle \psi \left|E_{k}\right|\psi \rangle$, and the post-interaction state is fixed by an associated set of Kraus operators $\left\{M_{k}\right\}$ with $E_{k}\;={\;M}_{k}^{†}M_{k}$, via $\left|\psi \rangle \mapsto M_{k}\right|\psi \rangle /∥M_{k}|\psi \rangle ∥$. Projective measurement is the special case $E_{k}\;=\;P_{k}$. Positive operator-valued measures let the model represent weak or noisy interactions in which superposition is only partly resolved, a flexibility we use when discussing decoherence in Section 3.2.

### 2.3. Density-Operator Representation and Decoherence

A single firm in a pure superposition is described by the density operator $\rho \;=\;\left|\psi \rangle \langle \psi \right|$. To represent a population of firms in various states, or a single firm whose phase information has partly degraded, we use a general density operator, Equation (3)

(3) $$\rho =\sum_{\mu }w_{\mu }\left|\psi_{\mu }\rangle \langle \psi_{\mu }\right|,\;\;w_{\mu }\;\geq \;0,\;\;\sum_{\mu }w_{\mu }=1,$$

a positive semidefinite operator with unit trace. In the strategy basis its entries split into two kinds with distinct economic meaning. The diagonal entries $p_{i}:=\rho_{\mathrm{ii}}=\;\langle s_{i}\left|\rho \right|s_{i}\rangle$ are the populations: $p_{i}$ is the share of the population that would realize strategy $s_{i}$ upon interaction, the quantity that feeds replicator dynamics. The off-diagonal entries $\rho_{\mathrm{ij}}$ with i≠j are the coherences: they measure the degree to which the population maintains genuine superposition between strategies $s_{i}$ and $s_{j}$, as opposed to a classical mixture in which firms have already settled. A purely diagonal density operator is mathematically identical to a classical probability distribution over pure strategies. Nonzero coherences are the signature of unresolved strategic superposition at the population level.

Interaction with an environment, including observation by competitors and the partial commitments of a market, suppresses coherences while leaving populations intact. This is decoherence, and in the theory of open quantum systems it is generated by a dissipator of Gorini–Kossakowski–Sudarshan–Lindblad form [32,33,34]. Taking the jump operators to be the strategy projectors $P_{k}\;=\;\left|s_{k}\rangle \langle s_{k}\right|$ with rates $\gamma_{k}\;\geq \;0$,

(4) $$D\left[\rho \right]=\sum_{k}\gamma_{k}\left(P_{k}\rho P_{k}-1/2\{P_{k},\rho \}\right)$$

a direct computation using $P_{k\;}^{2}=\;P_{k}$ gives $D{\left[\rho \right]}_{\mathrm{ij}}\;=\;0$ for i = j and $D{\left[\rho \right]}_{\mathrm{ij}}\;=\;-1/2\left(\gamma_{i}\;+\;\gamma_{j}\right)\rho_{\mathrm{ij}}$ for i≠j. Decoherence therefore acts as pure dephasing in the strategy basis: populations are untouched and each coherence decays at rate $\gamma_{\mathrm{ij}}:=1/2\left(\gamma_{i}\;+\;\gamma_{j}\right)$. Under D alone, ρ relaxes to its diagonal part $diag\left(p_{1},\ldots ,p_{n}\right)$, a classical distribution. The quantum-to-classical transition of a market is exactly this loss of strategic superposition: firms that maintain coherence keep their options open across strategies, while firms that have decohered have committed to a definite strategy in all but the sampling. The strategy basis is selected as the pointer basis precisely because it is the basis the environment monitors [19].

### 2.4. Replicator Preliminaries

Let $A\in R^{n\times n}$ be the payoff matrix of a symmetric game, with $A_{\mathrm{ij}}$ the payoff to strategy $s_{i}$ against strategy $s_{j}$. For a population state $p\in \Delta^{n-1}$, the fitness of strategy $s_{i}$ is $f_{i}\left(p\right)\;=\;{\left(\mathrm{Ap}\right)}_{i}\;=\;\sum_{j}A_{\mathrm{ij}}p_{j}$, and the mean fitness is $\varphi \left(p\right)\;=\;p\cdot \mathrm{Ap}\;=\;\sum_{i}p_{i}f_{i}\left(p\right)$. The replicator equation is

(5) $${\dot{p}}_{i}={\;p}_{i}\left(f_{i}\left(p\right)-\varphi \left(p\right)\right),\;\;i=1,\ldots ,n$$

Summation of Equation (5) gives $\sum_{i}{\dot{p}}_{i}=\;\varphi -\varphi \;=\;0$, so the simplex is invariant, and its faces are invariant as well. Interior rest points solve $f_{i}\left(p\right)\;=\;\varphi \left(p\right)$ for all i with $p_{i}>0$, equalizing the fitness of all strategies present [6,21,22].

### 2.5. The Strategic Master Equation

The full dynamics on the density operator combines three processes acting on potentially different timescales [34]. The first is coherent deliberation, an internal weighing of options that preserves superposition and is generated by a Hermitian deliberation Hamiltonian H through the von Neumann term $-i\left[H,\rho \right]$. Its matrix elements carry economic content: the diagonal $H_{\mathrm{ii}}$ represents a utility or payoff bias on the pure strategy $s_{i}$, while the off-diagonal $H_{\mathrm{ij}}$ is the propensity for coherent deliberative transition, or attention shift, between strategies $s_{i}$ and $s_{j}$. The second is decoherence in the strategy basis, defined in Section 2.3, which suppresses coherences while leaving populations intact. The third is selection, which reshapes the population frequencies according to differential fitness. We represent selection by a replicator superoperator R, defined in the strategy basis by

(6) $$R{\left[\rho \right]}_{\mathrm{ij}}=1/2\left(f_{i}\left(p\right)+f_{j}\left(p\right)-2\varphi \left(p\right)\right)\;\rho_{\mathrm{ij}},\;\;p_{k}={\;\rho }_{\mathrm{kk}},$$

with $f_{i}$ and φ the fitness and mean fitness of Section 2.4. The off-diagonal form assigns to each coherence $\rho_{\mathrm{ij}}$ the average of the two selection gradients, $1/2\left[\left(f_{i}-\varphi \right)\;+\;\left(f_{j}-\varphi \right)\right]$, which is the Hermitian lift of the replicator field consistent with the amplitude dynamics introduced above.

**Remark 1** **(Uniqueness of the lift).** *The off-diagonal action of* R *is not the only Hermitian, trace-preserving extension of the replicator field. The diagonal is fixed by* R[ρ]_ii_ = (f_i_ − φ)p_i_, *but any real symmetric multiplier* R[ρ]_ij_ = μ_ij_ ρ_ij_ *with* μ_ij_ = μ_ji_ *preserves Hermiticity and trace and reproduces the same population equation, so an infinite family of lifts exists, including* μ_ij_ = 0. The choice μ_ij_ = ½(f_i_ + f_j_ − 2φ) *is singled out by consistency with the amplitude representation of* Section 3.1*: on a pure state* ρ_ij_ = c_i_$\bar{c_{j}}$, *the amplitude flow* ċ_i_ = ½(f_i_ − φ)c_i_ *gives* d(c_i_$\bar{c_{j}}$)/dt = ½(f_i_ + f_j_ − 2φ)ρ_ij_, *so ours is the unique lift whose restriction to pure states coincides with the square-root selection dynamics. Alternative lifts generate different coherence dynamics and different finite-decoherence equilibria and stability, but all reduce to the same classical replicator equation in the strong-decoherence limit, where the coherences vanish; the distinction is observable only in the finite-*γ *regime.*

Combining the three contributions, the strategic master equation governing the joint evolution of populations and coherences is

(7) $$\dot{\rho \;}=\;-\;i\;\left[H,\rho \right]+D\left[\rho \right]+R\left[\rho \right].$$

Here $-i\left[H,\rho \right]$ generates coherent deliberation and interference, $D\left[\rho \right]$ drives the quantum-to-classical transition toward the strategy (pointer) basis, and $R\left[\rho \right]$ implements evolutionary selection. The dynamics is nonlinear.

## 3. Results

### 3.1. Amplitude Representation of the Replicator Field

The first result places quantum normalization and evolutionary selection on a single geometric footing. Writing the amplitude moduli as $r_{i}\;=\sqrt{p_{i}}$, the replicator equation on the simplex is carried to a flow on the positive orthant of the unit sphere $S_{+}^{n-1}$,

(8) $${\dot{r}}_{i}=1/2\;r_{i}\left(f_{i}\left(r⊙r\right)-\varphi \left(r⊙r\right)\right),\;\;{\left(r⊙r\right)}_{i}:=r_{i}^{2},$$

where ⊙ denotes the Hadamard (entrywise) product, defined for vectors u, v ∈ ℝ^n^; by (u⊙v)_i_ = u_i_v_i_. Thus r⊙r is the vector of componentwise squares, (r⊙r)_i_ = r_i_^2^ = p_i_, which recovers the population state p from the amplitude moduli r, so that f_i_(r⊙r) and φ(r⊙r) denote the fitness and mean fitness evaluated at that induced distribution. The correspondence is a bijection between simplex and sphere trajectories that preserves (Proposition A1). The map $r_{i}\;=\sqrt{p_{i}}$ is, up to a constant factor, the isometry between the simplex with the Shahshahani metric and the sphere with the round metric, since $\sum_{i}d\;r_{i}^{2}\;=1/4\sum_{i}d\;p_{i}^{2}/p_{i}$ [6,35,36]. For games with symmetric payoff matrix the amplitude flow is the Shahshahani gradient ascent. The economic content is that selection acts directly on the quantum amplitudes constrained to the normalization sphere, with fitter strategies acquiring larger amplitude, while the phases remain inert under population-level selection.

### 3.2. Structural Properties of the Replicator Superoperator

The selection superoperator $R{\left[\rho \right]}_{\mathrm{ij}}\;=1/2\left(f_{i}\left(p\right)\;+\;f_{j}\left(p\right)\;-\;2\varphi \left(p\right)\right)\rho_{\mathrm{ij}}$ reproduces the classical replicator field on the diagonal, $R{\left[\rho \right]}_{\mathrm{ii}}\;=\;\left(f_{i}\;-\;\varphi \right)p_{i}$; preserves Hermiticity; and annihilates the trace, so the density operator retains unit trace. Its action on coherences is the natural Hermitian lift of the replicator field, consistent with the amplitude dynamics of Section 3.1. The evolution preserves positivity and unit trace for arbitrary n and independently of the decoherence rates, so ρ remains a valid density operator throughout (Proposition A3, Appendix A), and its spectrum stays in [0,1]. Complete positivity in the Gorini–Kossakowski–Sudarshan–Lindblad sense is a property of linear generators and is not the operative criterion here, since R is state-dependent and the full evolution is not a linear quantum dynamical semigroup; the linear part −i[H,·] + D is completely positive, while for the nonlinear selection part the relevant and now established property is positivity of the single-population state.

### 3.3. Emergence of Classical Replicator Dynamics in the Decoherence Limit

The central analytical result is that classical evolutionary dynamics is the strong-decoherence limit of the strategic master equation. With $\Gamma \;={\;\min}_{i\neq j}\gamma_{\mathrm{ij}}$, adiabatic elimination of the coherences yields, to leading order, the closed replicator equation for the populations,

(9) $${\dot{p}}_{i}=p_{i}\left(f_{i}\left(p\right)-\varphi \left(p\right)\right)+O\;\left(\frac{∥H{∥}^{2}}{\Gamma }\right)$$

(Theorem A1).

### 3.4. Stability of Evolutionarily Stable Strategies

For an interior evolutionary stable strategy (ESS) $x^{*}$ [4], the cross-entropy $V\left(p\right)=\sum_{i}x_{i}^{*}\ln\left(x_{i}^{*}/p_{i}\right)$ is a strict local Lyapunov function for the replicator dynamics. By Theorem A1 this Lyapunov function governs the diagonal of the strategic master equation in the strong-decoherence regime, so interior ESS attracts the population once decoherence dominates. The natural candidate for the full quantum dynamics is the quantum relative entropy $S\left(\rho^{*}\|\rho \right)\;=\;\mathrm{Tr}\;\rho^{*}\left(\ln\rho^{*}\;-\;\ln\rho \right)$, which reduces to V on diagonal states and is monotone nonincreasing under the decoherence part of the dynamics by Lindblad’s theorem [37]; the nonlinear selection term is left for separate treatment.

### 3.5. Context Dependence and Interference

Strategy realization is shown to be basis-sensitive. For a rotated measurement basis the realized outcome probabilities are

(10) $$q_{k}=\sum_{i}{\left|U_{\mathrm{ki}}\right|}^{2}p_{i}+\sum_{i\neq j}U_{\mathrm{ki}}\;\bar{U_{\mathrm{kj}}}\;\bar{c_{i}}\;c_{j}$$

where the first sum is the classical prediction and the second is an interference term depending on the relative phases of the amplitudes (Proposition A2). No classical mixture over pure strategies with the same marginals $p_{i}$ reproduces $q_{k}$ for all rotations. This is the formal counterpart of framing and context effects [9] and of the order and disjunction effects that motivate quantum decision theory [8,11], and it is a feature classical evolutionary game theory cannot represent.

Two points of interpretation follow. The strategy basis itself is operationally fixed: it is the basis of committed, realized strategies that market interaction renders definite, selected as the pointer basis by environmental monitoring (Section 2.3). The rotation U in (10) is not a free internal parameter but is set by the interaction context, a framing, an institutional arrangement, or an information-disclosure regime, with each context fixing a specific U; operationally, U is recovered for a given context from the discrepancy between the realized distribution $q_{k}$ and the strategy-basis marginals $p_{i}$. The context-to- U map is an empirical input rather than an object derived from first principles, exactly as behavioral economics measures framing effects rather than deriving them, and this does not reduce predictive power, since $q_{k}$ is fully determined once ρ and the context are fixed. The construction is moreover falsifiable: by Proposition A2, no classical mixture with the same marginals reproduces $q_{k}$ for all U, so varying the context and measuring the realized distribution discriminates the superposition account from any classical-mixture account with identical marginals.

### 3.6. Numerical Results for the Two-Strategy Market Game

We instantiate the framework in the Hawk–Dove game, the canonical two-strategy contest over a resource of value V won or shared at cost C. Writing $s_{1}$ = Hawk and $s_{2}$ = Dove, the payoff to the focal player is A = $\left(\begin{array}{cc} (V-C)/2 & V\\ 0 & V/2 \end{array}\right)$ which for V = 2, C = 3 gives A = $\left(\begin{array}{cc} -0.5 & 2\\ 0 & 1 \end{array}\right)$. The classical replicator dynamics of this game has a unique interior evolutionarily stable strategy at the Hawk share $p^{*}=V/C=2/3$, where the two strategies have equal fitness. The population is the qubit density operator ρ =$\;\left(\begin{array}{cc} p & c\\ \bar{c} & 1-p \end{array}\right)$ with $p\;=\;p_{1}$ the Hawk share and $c\;={\;\rho }_{12}$ the single coherence. The deliberation Hamiltonian is the off-diagonal coupling $H\;=\;\Delta \left(|s_{1}\right\rangle \left\langle s_{2}|+|s_{2}\right\rangle \left\langle s_{1}|\right)$, so that ∥H∥=Δ sets the strength of coherent deliberation, and decoherence acts on the coherence at rate γ. In the reduced dynamics, the real part of c decouples and decays, so the stationary state is governed by p and the imaginary part of c.

**Figure 1:**
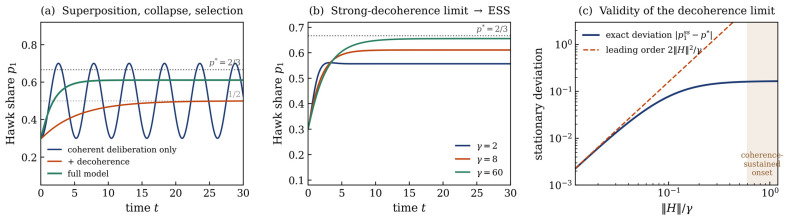
Joint dynamics of superposition, collapse, and selection in the Hawk–Dove game (V=2, C=3, interior ESS $p^{*}=2/3$). (**a**) Hawk share under coherent deliberation only, with added decoherence, and in the full model. (**b**) Full model for decoherence rates $\gamma \in \left\{2,8,60\right\}$, converging to the ESS. (**c**) Stationary deviation $|p_{1}^{\mathrm{ss}}-p^{*}|$ versus ∥H∥/γ, following the $2∥H{∥}^{2}/\gamma$ scaling of Theorem A1 and departing from it near ∥H∥/γ=1 (shaded). Time is dimensionless (see text).

Figure 1a fixes Δ = 0.6 and γ = 8 and contrasts three settings from a common initial Hawk share $p_{1}\;=\;0.30$. Under coherent deliberation alone (the Hamiltonian term only), the population executes undamped oscillations about p = 1/2 and never settles, showing that deliberation without commitment or selection cannot produce a determinate strategic outcome. Adding decoherence but no selection damps these oscillations to the fitness-blind value p = 1/2: the market resolves the superposition into a definite strategy, but, with absent selection, the resolved distribution carries no adaptive information. Only the full model, which adds replicator selection, drives the population toward the evolutionarily stable strategy. The three trajectories isolate the roles of the three generators and show that coherent deliberation, decoherence, and selection are each individually insufficient, the adaptive outcome requiring collapse and selection acting together.

Figure 1b fixes Δ = 0.6 and varies the decoherence rate $\gamma \in \left\{2,8,60\right\}$ in the full model, again from $p_{1}\;=\;0.30$. In every case, the population converges to a stationary Hawk share, and as γ increases this share rises toward the classical ESS $p^{*}\;=\;2/3$. The finite-γ equilibrium sits systematically below the classical value, displaced by an amount of order $\Delta^{2}/\gamma$ that shrinks as decoherence strengthens. This is the numerical content of Theorem A1: strong decoherence recovers the classical replicator equilibrium, while sustained coherence at finite γ produces a quantifiable deviation from it. The decoherence rate thus acts as the control parameter interpolating between the quantum-corrected and the classical evolutionary outcome.

Figure 1c probes the range of validity of this reduction. Holding γ = 8 fixed and varying ∥H∥, the stationary deviation of the Hawk share from $p^{*}$ follows the leading-order $2∥H{∥}^{2}/\gamma$ prediction of Theorem A1 while ∥H∥/γ is small, and departs from it as ∥H∥/γ  approaches unity, where the coherences are no longer slaved to the populations and the system enters the coherence-sustained regime.

Time is dimensionless throughout: the payoff entries, the coupling ∥H∥, and the decoherence rate γ are all measured as rates in the same inverse-time units, so one unit on the horizontal axis corresponds to the inverse of the fitness scale that sets the selection timescale.

## 4. Discussion

Quantum effects are transient and corrective, except in a coherence-sustained regime. The results draw a clean line. In the decoherence-dominated regime, long-run population outcomes are classical evolutionarily stable strategies (Theorem A1 and Section 3.4), and quantumness appears only as transient, individual-level deliberation and as a finite-γ correction to equilibria. But the correction is real, and it grows as coherent coupling strengthens relative to decoherence and to the selection gradients. When that ratio is large, the classical ESS can be substantially displaced or destabilized, and the population can sustain coherent, oscillatory behavior rather than relaxing to a classical rest point.

The construction is complementary to, and distinct from, both established lines. The quantum games program quantizes the act of play, with actors choosing unitary operations on entangled states and the analysis centered on quantum equilibria [17,18,19]. The present model keeps payoffs and the act of play classical and locates the novelty earlier, in the pre-commitment strategic state and its collapse at interaction, then couples that collapse to population dynamics. Quantum decision theory models the single choice of a single agent and explains anomalies of individual choice through interference [10,11,12,13,14,15,16]. The present model embeds that machinery in evolutionary dynamics, so that interference at the decision layer propagates into selection at the population layer, as Proposition A2 makes explicit.

Strategic superposition gives a formal model of an indeterminate pre-decision state that does not require positing a hidden optimum the actor approximates. This complements the bounded-rationality tradition [38], which abandons the optimizing ideal without supplying a positive account of the unsettled state that precedes choice. Within the Homo Evolutivus program, the superposition is the mathematical content of constitutive self-opacity: the firm’s strategy is constitutively indefinite, and selection operates on the squared amplitudes that collapse produces, reshaping the distribution of strategic indeterminacy over evolutionary time.

Four limitations are salient. The selection superoperator R is phenomenological; the evolution preserves positivity for arbitrary n (Proposition A3), while complete positivity is not the operative criterion for a nonlinear generator, and a fuller operator-theoretic characterization of admissible nonlinear selection superoperators remains open. The model treats a single population, so entanglement between interacting firms, which would let their strategic choices be correlated in ways no independent mixture allows, is not represented; its generalization is sketched below and left for a dedicated study. The payoff structure is classical, so the framework does not address quantized payoffs. The deliberation Hamiltonian is posited rather than derived, as is standard in quantum decision theory; a microfoundation from an explicit optimization or information-processing principle remains open, though the harmonic component of the potential-harmonic decomposition of games [39] offers a candidate route, since it generates precisely the conservative, cyclic dynamics that a Hamiltonian term produces. Its elements are in principle identifiable from observables, the deliberation frequency scaling with ∥H∥, the finite-decoherence displacement with $\Delta^{2}/\gamma$, and the off-diagonal phases entering through the interference term of Proposition A2, but full structural identification of both H and the decoherence rate from market data remains an open measurement problem.

To indicate the generalization, on $H_{A}⊗H_{B}$ the strategic master equation becomes ${\dot{\rho }}_{\mathrm{AB}}=-i\left[H_{\mathrm{AB}},\rho_{\mathrm{AB}}\right]+D_{\mathrm{AB}}\left[\rho_{\mathrm{AB}}\right]+R_{\mathrm{AB}}\left[\rho_{\mathrm{AB}}\right]$, with $H_{\mathrm{AB}}=H_{A}⊗I+I⊗H_{B}+H_{\mathrm{int}}$, the interaction term $H_{\mathrm{int}}$ generating entanglement, $D_{\mathrm{AB}}$ local dephasing of each party in its own strategy basis with jump operators $P_{k}^{A}⊗I$ and $I⊗P_{l}^{B}$, and $R_{\mathrm{AB}}$ the selection superoperator with correlated joint fitness, $R_{\mathrm{AB}}{\left[\rho \right]}_{\left(\mathrm{ij}\right),\left(\mathrm{kl}\right)}=\frac{1}{2}\left(f_{\mathrm{ij}}+f_{\mathrm{kl}}-2\varphi \right)\rho_{\left(\mathrm{ij}\right),\left(\mathrm{kl}\right)}$, where $p_{\mathrm{ij}}=\left\langle s_{i}s_{j}|\rho_{\mathrm{AB}}|s_{i}s_{j}\right\rangle$ and $\varphi =\sum p_{\mathrm{ij}}f_{\mathrm{ij}}$. The non-product entries encode cross-firm strategic correlations that no independent mixture reproduces. Positivity is preserved by the same argument as in the single-population case, since the tangent-cone condition of Proposition A3 is dimension and basis-independent and $R_{\mathrm{AB}}$ again vanishes on the kernel of $\rho_{\mathrm{AB}}$. In the strong-decoherence limit, the joint dynamics reduces to multipopulation replicator dynamics, whose interior rest points coincide with Nash equilibria, so the relevant stability notion is evolutionary stability for asymmetric games [40] rather than the single-population evolutionarily stable strategy. Full tractability does not extend to large strategic networks, however, since the joint operator grows exponentially in the number of firms, so mean-field or low-entanglement approximations are required, which we leave for future work.

### 4.1. Managerial Implications

Although the construction is abstract, three of its features carry directly into managerial reasoning about competitive strategy. First, the model formalizes the pre-entry position of a firm as constitutively indeterminate rather than as a decided plan awaiting execution, so that a firm’s realized competitive posture crystallizes at the moment of market interaction rather than being fixed beforehand. For a manager, this reframes early market engagements as the events that select a strategy from a still-open set, which places a premium on the framing and timing of the first competitive encounter rather than on the illusory precision of a pre-committed plan. Second, the context dependence established in Proposition A2 implies that the same underlying strategic disposition can realize different strategies depending on the basis in which interaction is resolved, so that the order and framing effects already documented at the level of individual choice [7,9] propagate to the level of realized firm behavior. Managers who control the frame of an initial interaction, through the choice of channel, reference class, or competitive comparison, therefore exert a measurable influence on which strategy is realized, an influence invisible to any account that fixes strategies before play. Third, the order Δ^2^/γ displacement of the evolutionary equilibrium quantifies when classical equilibrium reasoning is safe and when it misleads. In mature markets with frequent, sharply resolved interactions, decoherence dominates and the classical evolutionarily stable strategy is a reliable planning benchmark; in novel or thinly traded markets, where deliberation is sustained under ambiguity and decoherence is weak, the classical benchmark is displaced by a predictable amount and can be destabilized entirely, so that reliance on standard equilibrium forecasts is least warranted precisely where managerial stakes are highest. The coherence-sustained regime, finally, describes markets in which competitive behavior does not settle to a fixed configuration but sustains structural oscillation, so that the absence of convergence is a property of the environment rather than a symptom of mismanagement.

### 4.2. Applications and Societal Relevance

The framework is intended to apply wherever strategic actors commit under genuine pre-decision ambiguity and where the frame of interaction shapes the outcome. Market entry into novel categories, such as platform launches, deep-technology ventures, and newly created product classes, is the natural domain, since there the entering firm has often not settled on a strategy in any operational sense and the classical premise of a definite pre-interaction plan is least tenable. The documented interference, order, and framing effects that motivate the construction [7,9] give the model an empirical anchor at the individual level; embedding that machinery in population dynamics extends it to the aggregate behavior of firms and offers, through the observable signatures collected in the Conclusion, a route to testing the framework against market or experimental data rather than treating it only as an interpretive lens.

Beyond these applications, the construction bears on how economic prediction is used in public decision-making. If pre-decision strategic states are constitutively indeterminate and their realization is context-dependent, then policy and market design premised on stable, predictable rational-actor equilibria are most fragile in exactly the emerging and high-stakes markets, competition in digital platforms and artificial-intelligence services among them, where prediction is most consequential. The framework does not license stronger normative claims than its abstraction supports, but it does supply a disciplined reason for epistemic humility about equilibrium forecasts in such settings, and it locates that humility in a formal structure rather than in a general appeal to complexity. In this respect, it contributes to a behavioral foundation for economics that takes the failures of classical probability in human choice seriously without abandoning the analytical tractability of replicator dynamics, which is the aim of the Homo Evolutivus program within which the model sits.

## 5. Conclusions

We have constructed a mathematical foundation that integrates quantum decision theory with evolutionary game theory and replicator dynamics. An economic actor before market entry is modeled as a superposition of pure strategies in a Hilbert space; the moment of interaction is a measurement that collapses the superposition onto a realized strategy with Born-rule probabilities; and these probabilities, carried by the diagonal of a density operator, evolve under a strategic master equation whose three superoperators represent coherent deliberation, decoherence in the strategy basis, and replicator selection. The amplitude representation places quantum normalization and evolutionary selection on a common geometric footing through the square-root map to the sphere. The decoherence-limit theorem shows that classical replicator dynamics is the strong-decoherence limit of the quantum strategic dynamics, with an explicit error bound. Context dependence distinguishes superposed strategies from any classical mixture with the same marginals. And the worked two-strategy game demonstrates that coherent coupling displaces the evolutionary equilibrium from the classical evolutionarily stable strategy by a quantity of order $\Delta^{2}/\gamma$, recovering the stable strategy as decoherence dominates. The construction formalizes constitutive self-opacity and connects bounded rationality to the interference structure of quantum probability. The bifurcation between the decohered-classical regime and the coherence-sustained regime, together with entanglement between firms, defines the agenda for the installments that follow.

The present work opens several concrete lines for future research. The first is a dedicated bifurcation analysis of the coherence-sustained regime, where the coherences are no longer slaved to the populations and the leading-order reduction of Theorem A1 ceases to be uniform, so that the transition between the decohered-classical regime and the oscillatory regime becomes an object of study in its own right. The second is the entangled bipartite dynamics sketched in Section 4, in which an interaction Hamiltonian couples two populations and generates cross-firm strategic correlations that no independent mixture reproduces; a full treatment requires the mean-field and low-entanglement approximations needed once the joint operator grows exponentially in the number of firms. The third is empirical: the observable signatures identified here, namely the deliberation frequency scaling with ‖H‖, the order Δ^2^/γ displacement of equilibria, and the context dependence of realized choice established in Proposition A2, are designed to make the deliberation Hamiltonian and the decoherence rate identifiable from market or experimental data, turning the construction into a testable rather than a merely interpretive framework. The fourth is structural generalization beyond symmetric two-strategy games to asymmetric contests and larger strategy sets, where evolutionary stability must be assessed for asymmetric games rather than through the single-population evolutionarily stable strategy. Together with the managerial and applied questions discussed above, these define the agenda for the installments that follow.

## Data Availability

No new data were created or analyzed in this study. Data sharing is not applicable to this article.

## Nomenclature

The following symbols/operators are used in this manuscript:

$H\;≅\;C^{n}$	strategy Hilbert space
$S=\left\{s_{1},\ldots ,s_{n}\right\}$	set of pure strategies
$\left.|s_{i}\right\rangle$	strategy (pointer) basis state for pure strategy $s_{i}$
$\left.|\psi \right\rangle$	pre-interaction strategic superposition
$c_{i}=r_{i}e^{i\theta_{i}}$	probability amplitude of $s_{i}$ (modulus $r_{i},$ phase $\theta_{i}$)
$p_{i}=|c_{i}{|}^{2}=\rho_{\mathrm{ii}}$	realization probability/population share of $s_{i}$
ρ	population density operator
$\rho_{\mathrm{ij}}\;\left(i\neq j\right)$	coherence between strategies $s_{i}$ and $s_{j}$
$\left.P_{i}=|s_{i}\right\rangle \left\langle s_{i}|\right.$	strategy-basis projector/dephasing jump operator
$E_{k},\;M_{k}$	POVM elements and associated Kraus operators
$H,\;H_{\mathrm{ij}}$	deliberation Hamiltonian (Hermitian) and its matrix elements
∥H∥	operator norm of H; coherent coupling scale (Δ in the qubit game)
$D\left[\rho \right]$	decoherence (dephasing) dissipator
$\gamma_{k},\;\gamma_{\mathrm{ij}}=\frac{1}{2}\left(\gamma_{i}+\gamma_{j}\right),\;\Gamma =\min_{i\neq j}\gamma_{\mathrm{ij}}$	decoherence rate of $s_{k},$ coherence decay rate, minimal rate
$A,\;A_{\mathrm{ij}}$	payoff matrix and its entries
$f_{i}\left(p\right)={\left(\mathrm{Ap}\right)}_{i},\;\varphi \left(p\right)=p\cdot \mathrm{Ap}$	fitness of $s_{i}$ and mean fitness
$R\left[\rho \right]$	replicator selection superoperator
$U,\;U_{\mathrm{ki}}$	measurement-basis rotation (context) and its entries
$q_{k}$	realized outcome probabilities in the rotated basis
$\Delta^{n-1},\;S_{+}^{n-1}$	probability simplex and positive orthant of the unit sphere
$r_{i}=\sqrt{p_{i}}$	amplitude modulus (square-root coordinate)
$V\left(p\right),\;S\left(\rho^{*}∥\rho \right)$	cross-entropy Lyapunov function and quantum relative entropy
$x^{*},\;\lambda$	interior ESS and replicator-Jacobian spectral gap
V, C	Hawk–Dove resource value and contest cost

## Appendix A

This appendix collects the formal statements of the two propositions and the theorem used in the main text, together with their proofs. The notation is that of Section 2 and Section 3.

**Proposition A1** **(Amplitude Representation).** *Let* $p\left(t\right)\in \mathrm{int}\Delta^{n-1}$ *solve the replicator equation, and define the amplitude moduli* $r_{i}\left(t\right)=\sqrt{p_{i}\left(t\right)}\geq 0$ *. Then,* $r\left(t\right)$ *lies on the positive orthant of the unit sphere* $S_{+}^{n-1}=\{r:\sum_{i}r_{i}^{2}=1,\;r_{i}\geq 0\}$ *and satisfies*

$${\dot{r}}_{i}=1/2\;r_{i}\left(f_{i}\left(r⊙r\right)-\varphi \left(r⊙r\right)\right),\;\;{\left(r⊙r\right)}_{i}:=r_{i}^{2}.$$

*Conversely, any solution of this equation with* $r\in S_{+}^{n-1}$ *projects, via* $p_{i}=r_{i}^{2}$ *, to a solution of the replicator equation, and* $\sum_{i}r_{i}^{2}=1$ *is preserved.*

**Proof of** **Proposition A1.** From $p_{i}=r_{i}^{2}$ we have ${\dot{p}}_{i}=2r_{i}{\dot{r}}_{i}$. Substituting the replicator right-hand side gives $2r_{i}{\dot{r}}_{i}=r_{i}^{2}\left(f_{i}-\varphi \right)$, hence ${\dot{r}}_{i}=1/2r_{i}\left(f_{i}-\varphi \right)$ wherever $r_{i}>0$; the boundary $r_{i}=0$ is invariant because the corresponding simplex face is invariant. Normalization follows from $\frac{d}{\mathrm{dt}}\sum_{i}r_{i}^{2}=\frac{d}{\mathrm{dt}}\sum_{i}p_{i}=0$. The converse reverses these steps. □

**Theorem A1** **(Decoherence Limit).** *Fix* H *and* A *, and write* $\Gamma =\min_{i\neq j}\gamma_{\mathrm{ij}}$ *. As* Γ→∞ *, adiabatic elimination of the coherences in the strategic master equation yields, to leading order, the closed replicator equation for the populations* $p_{i}=\rho_{\mathrm{ii}}$ *,*

$${\dot{p}}_{i}=p_{i}\left(f_{i}\left(p\right)-\varphi \left(p\right)\right)+O\;\left(\frac{∥H{∥}^{2}}{\Gamma }\right).$$

*Classical replicator dynamics is thus recovered exactly in the limit of strong decoherence. The estimate is uniform on finite intervals of the slow time and on compact subsets of the simplex interior on which the reduced replicator field is normally hyperbolic. If the reduced dynamics possesses a hyperbolic interior attractor* x* *(an evolutionarily stable strategy with replicator Jacobian spectral gap* λ > 0*,* cf. Section 3.4*), the estimate holds uniformly in time, with a constant of order* 1/λ.

**Proof of** **Theorem A1.** Split ρ into its diagonal part and its off-diagonal part. For i≠j, the coherence obeys

$${\dot{\rho }}_{\mathrm{ij}}=-\;i\;{\left[H,\rho \right]}_{\mathrm{ij}}-\gamma_{\mathrm{ij}}\;\rho_{\mathrm{ij}}+1/2\left(f_{i}+f_{j}-2\varphi \right)\rho_{\mathrm{ij}}$$

The part of $-i{\left[H,\rho \right]}_{,}$ sourced by the diagonal is $-i\;H_{\mathrm{ij}}\left(p_{j}-p_{i}\right)$. Setting ${\dot{\rho }}_{\mathrm{ij}}\approx 0$ and retaining the dominant source gives the quasi-steady coherence

$$\rho_{\mathrm{ij}}^{\mathrm{ss}}\approx \frac{-\;i\;H_{\mathrm{ij}}\left(p_{j}-p_{i}\right)}{\gamma_{\mathrm{ij}}-1/2\left(f_{i}+f_{j}-2\varphi \right)}=O\;\left(\frac{∥H∥}{\Gamma }\right).$$

The diagonal obeys ${\dot{\rho }}_{\mathrm{ii}}=-i{\left[H,\rho \right]}_{\mathrm{ii}}+R{\left[\rho \right]}_{\mathrm{ii}}$, where $R{\left[\rho \right]}_{\mathrm{ii}}=p_{i}\left(f_{i}-\varphi \right)$ is the replicator term. The Hamiltonian contribution to the diagonal is -iH,ρii=2∑kImHik ρik¯, which is real and, since each $\rho_{\mathrm{ik}}=O\left(∥H∥/\Gamma \right)$ by the quasi-steady-state estimate, is of order $∥H{∥}^{2}/\Gamma$. Hence, ${\dot{p}}_{i}=p_{i}\left(f_{i}-\varphi \right)+O\left(∥H{∥}^{2}/\Gamma \right)$. □

**Remark** **A1 (Range of validity).** *The quasi-steady elimination requires the coherence-damping rate Γ to dominate the selection gradients, the regime in which the coherence subsystem is normally hyperbolic and attracting, the same regime as Proposition 3. The reduction is then a Tikhonov-Fenichel singular perturbation, valid uniformly on finite slow-time intervals and, near a hyperbolic ESS, uniformly in time through the Lyapunov function of* Section 3.4*. The constant degrades as the spectral gap* λ → 0*, that is, near bifurcations of the replicator dynamics and when* ‖H‖/Γ *ceases to be small; there the coherences are no longer slaved and the leading-order reduction is not uniformly valid. This coherence-dominated regime, where the quantum corrections become non-perturbative, is left for separate treatment and is the subject of the bifurcation analysis announced in* Section 5*.*

**Proposition A2** **(Context Dependence).** *Let* $\left|\psi \rangle =\sum_{i}c_{i}\right|s_{i}\rangle$ *with realization probabilities* $p_{i}={\left|c_{i}\right|}^{2}$ *in the strategy basis. Suppose interaction occurs instead in a rotated basis* $\left\{\right|\phi_{k}\rangle \}$ *with* $\left|\phi_{k}\rangle =\sum_{i}U_{\mathrm{ki}}\right|s_{i}\rangle$ *for a unitary* U *. Then, the realized outcome probabilities are*

qk=⟨ϕkψ⟩|=2∑i,jUki Ukj¯ ci¯ cj=∑i |Uki|2pi+∑i≠jUki Ukj¯ ci¯ cj.

*The second sum is an interference term that depends on the relative phases of the amplitudes. No classical mixture over pure strategies with the same marginals* $p_{i}$ *reproduces* $q_{k}$ *for all rotations* U.

**Proof of** **Proposition A2.** Since $\left|\phi_{k}\rangle =\sum_{i}U_{\mathrm{ki}}\right|s_{i}\rangle$, we have $\langle \phi_{k}|\psi \rangle =\sum_{i}\bar{U_{\mathrm{ki}}}\;c_{i}$, so that $q_{k}=\left|\langle \phi_{k}\right|\psi \rangle {|}^{2}=\sum_{i,j}U_{\mathrm{ki}}\;\bar{U_{\mathrm{kj}}}\;\bar{c_{i}}\;c_{j}$ after relabeling the summation indices. Separating the diagonal i=j from the off-diagonal i≠j gives $\sum_{i}{\left|U_{\mathrm{ki}}\right|}^{2}p_{i}$, the classical mixing of the marginals through the weights ${\left|U_{\mathrm{ki}}\right|}^{2}$, plus the interference term $\sum_{i\neq j}U_{\mathrm{ki}}\;\bar{U_{\mathrm{kj}}}\;\bar{c_{i}}\;c_{j}$, whose summands depend on the relative phases through $\bar{c_{i}}\;c_{j}=r_{i}r_{j}e^{i\left(\theta_{j}-\theta_{i}\right)}$. A classical mixture over pure strategies is determined entirely by its marginals $p_{i}$ and yields only the diagonal term for every U ; hence, no such mixture reproduces $q_{k}$ for all rotations U. □

**Proposition **A3** (Positivity Preservation).** *Let* ρ(t) *evolve under the strategic master Equation (7) with* H *Hermitian, dephasing dissipator* D *of the form (4) with rates* γₖ ≥ 0*, and replicator superoperator* R *of the form (6). If* ρ(0) *is a density operator, then* ρ(t) ≥ 0 *and* Tr ρ(t) = 1 *for all* t ≥ 0*, for every dimension* n *and independently of the rates* γ_k_*. Consequently, the spectrum of* ρ(t) *remains in* [0,1].

**Proof of** **Proposition A3.** Hermiticity and unit trace are preserved: each generator preserves Hermiticity, Tr(-I[H,ρ]) = Tr D[ρ] = 0, and Tr R[ρ] = Σᵢ(fᵢ−φ)pᵢ = φ−φ = 0. The field F(ρ) = -I[H,ρ] + D[ρ] + R[ρ] is polynomial in the entries of ρ, hence locally Lipschitz, so by the Nagumo subtangentiality theorem the closed positive-semidefinite cone is forward-invariant provided ⟨v|F(ρ)|v⟩ ≥ 0 whenever ρ ≥ 0 and ⟨v|ρ|v⟩ = 0. Fix such ρ and v. Positivity of ρ and ⟨v|ρ|v⟩ = 0 give ρ|v⟩ = 0 and ⟨v|ρ = 0. Then, ⟨v|[H,ρ]|v⟩ = (⟨v|H)(ρ|v⟩)−(⟨v|ρ)(H|v⟩) = 0. With gᵢ = fᵢ−φ, the selection term is $\left\langle v\left|R\left[\rho \right]\right|v\right\rangle =\;½\;\Sigma ᵢ\;gᵢ\;\bar{v}ᵢ\;\left(\rho v\right)ᵢ\;+\;½\;\Sigma ⱼ\;gⱼ\;vⱼ\;\left(v†\rho \right)ⱼ\;=\;0$, since ρv = 0 and v†ρ = 0. Finally, with |wₖ⟩ = Pₖ|v⟩, ⟨v|D[ρ]|v⟩ = Σₖ γₖ ⟨wₖ|ρ|wₖ⟩ ≥ 0, using ${P_{k}}^{2}\;=\;Pₖ$ and ρ|v⟩ = ⟨v|ρ = 0 to annihilate the anticommutator. Hence, ⟨v|F(ρ)|v⟩ = ⟨v|D[ρ]|v⟩ ≥ 0, so the cone is invariant. Positivity with unit trace confines the eigenvalues to [0,1]. □
